# Does Left Atrial Size and E/e′ Predict Outcomes in Hypertrophic Cardiomyopathy?

**DOI:** 10.1111/echo.70345

**Published:** 2025-11-21

**Authors:** Brandon Tillson, Stephen Lord, Christopher Eggett

**Affiliations:** ^1^ Faculty of Medical Sciences Framlington Place Newcastle University England UK; ^2^ Freeman Hospital England UK

**Keywords:** atrial fibrillation, atrial flutter, cardiomyopathy, catheter ablation, echocardiography, hypertrophic, left atrium

## Abstract

**Background:**

Atrial arrhythmia is prevalent in patients with hypertrophic cardiomyopathy (HCM) and is prognostically deleterious. Catheter ablation (CA) has poor efficacy in HCM, making predictors of outcome valuable. Left atrial size is associated with morbidity and mortality, and E/e′ has been proposed. The current study evaluates whether these predict non‐paroxysmal atrial arrhythmia, interventions for atrial arrhythmia and all‐cause mortality.

**Methods:**

233 patients from the echocardiography database were included; patients with any mitral annular calcification were excluded. Medical records were reviewed for sample characteristics. Survival analysis was performed for left atrial diameter (LAD), left atrial volume index (LAVi, by area‐length method), and E/e′ with respect to endpoints, namely: non‐paroxysmal atrial arrhythmia; atrial intervention (composite of CA and pace‐and‐ablate); and all‐cause mortality. If LAD or LAVi were significant in multivariate analysis, ROC analysis and DeLong's test were performed to evaluate and compare their discriminative power.

**Results:**

Over a median follow‐up of 8 years (median age 53, 73% male), the overall prevalence of non‐paroxysmal atrial arrhythmia was 31%. In multivariate models, LAVi was predictive of non‐paroxysmal atrial arrhythmia and atrial intervention; LAD was only predictive of arrhythmia. LAVi demonstrated greater discriminative power for predicting atrial intervention. E/e′ was not predictive of any outcome. No echocardiographic variable predicted death in multivariate analysis.

**Conclusion:**

LAVi may have greater clinical utility compared to two‐dimensional measurements like LAD. Future work should clarify which measures of left atrial size are most appropriate in clinical practice.

## Graphical abstract

The current study followed 233 patients with hypertrophic cardiomyopathy, over a median follow‐up of 8 years. Left atrial size but not E/e' were predictive of outcomes related to atrial arrhythmia, and none were predictive of all‐cause mortality in multivariate analysis.

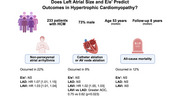

## 1 Introduction

Hypertrophic cardiomyopathy (HCM) is a primary myocardial disease arising from mutations in sarcomeric proteins [1, 2, 3]. It is the most common inherited cardiac condition, with a prevalence in adults of around 1:500 [4, 5]. Atrial fibrillation (AF) is common and associated with poorer cardiovascular and all‐cause mortality [6, 7, 8]. There is a distinct underlying anatomical substrate, with variable diastolic impairment, ventricular fibrosis [9, 10], and left atrial dilatation [10]. Catheter ablation (CA) unfortunately yields poor results in HCM [11, 12, 13, 14, 15]. The National Institute for Health and Care Excellence lists HCM as an absolute contraindication to CA in persistent AF [16]. However, CA is performed both anecdotally and in the literature [11, 12, 13, 14, 15] in selected cases, making predictors of outcome valuable.

The relationship between left atrial size and outcomes has been reported in HCM and is associated with the onset of AF [7, 8, 17, 18, 19], efficacy of CA [11, 13], and both cardiovascular and all‐cause mortality [20, 21, 22]. This is particularly true for left atrial diameter (LAD), with left atrial volume index (LAVi) less frequently reported. E/e′ may predict the onset of atrial arrhythmia [18].

The HCM Risk‐SCD calculator has been widely used in the risk stratification of patients, particularly in guiding ICD therapy [23]. The HCM‐AF score helps in assessing the risk of atrial fibrillation (AF) [17]. Both scores use LAD in addition to septal thickness and peak LVOT gradient.

There is heterogeneity in how LAVi is measured. The biplane method uses the method of disks in the apical two‐ and four‐chamber views to estimate volume. However, the area‐length method (using the same windows), although arguably simpler to perform, may overestimate volumes [24, 25]. Since 2020, the British Society of Echocardiography has included LAVi (by biplane method) in the minimum dataset [26]. Although LAD provides clinically meaningful information, it may be limited compared to more granular measurements like LAVi.

## 2 Materials and Methods

### 2.1 Study Design and Planning

**FIGURE 1:**
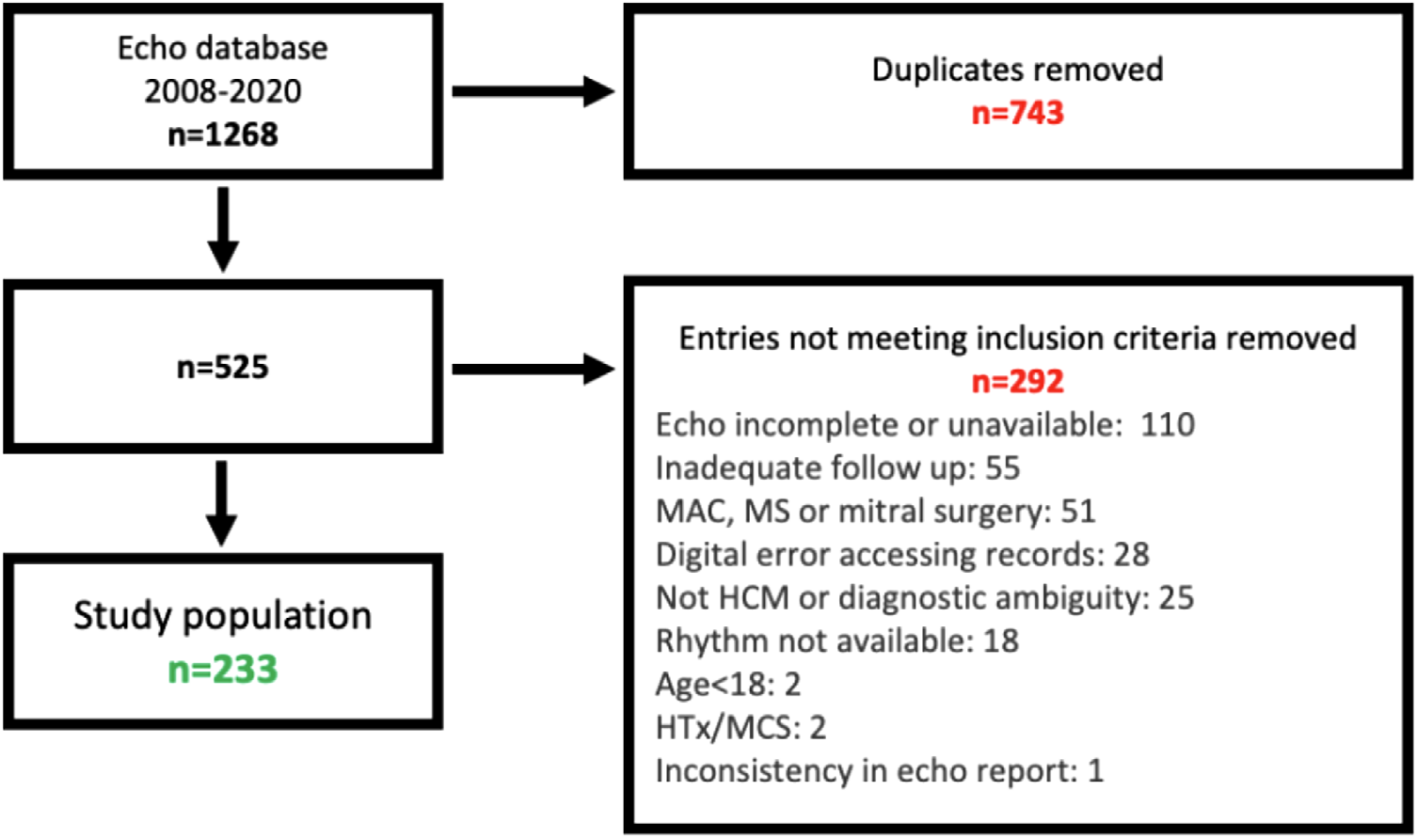
Flow diagram for study population.

A retrospective, observational, single‐center medical notes review spanning 2008–2020 was conducted. All patients were under follow‐up for HCM, at the Cardiothoracic Centre, Freeman Hospital, The Newcastle upon Tyne Hospitals NHS Foundation Trust, United Kingdom. From the echocardiography database, all patients where “cardiomyopathy, hypertrophic” was an indication were extracted. The final study population comprised *n* = 233 patients (Figure 1). Of the excluded patients, 17% were excluded because they had a history of mitral stenosis, mitral annular calcification, or mitral valve surgery.

Inclusion criteria are as follows:

- Resting transthoracic echocardiogram available at age ≥18;
- Septal thickness ≥15 mm on the index echocardiogram (if ≥13 mm, must be with family history or diagnosis documented in clinic letter);
- Rhythm documented at the index and the end of the follow‐up;
- Follow‐up for ≥18 months (to enable sufficient data collection).

Exclusion criteria are as follows:

- Any history of mitral stenosis, mitral annular calcification, or mitral valve surgery (focal mitral *leaflet* calcification was **not** excluded);
- Suggestion of diagnostic uncertainty for HCM;
- Heart transplantation or mechanical circulatory support previously or during the follow‐up.

### 2.2 Data Collection

For each patient, the index was the first resting transthoracic echocardiography report meeting inclusion criteria (thus, peak LVOT gradient was measured at rest, not Valsalva or during exercise). To establish sample characteristics, medical records were searched from 12 months before to 6 months after that point. The end of follow‐up was the final cardiology clinic letter, cardiology discharge summary or date of death (if unavailable, final clinical contact was used). Endpoints were established by comprehensively reviewing the medical records. Inclusion criteria necessitated a minimum follow‐up of 18 months.

For the echocardiographic variables, left ventricular outflow tract gradient was recorded as the peak measurement at rest, as explained above. Left ventricular systolic dysfunction (LVSD) was defined as an ejection fraction < 50% or qualitatively impaired left ventricular global systolic function of any severity. E/e' was the mean value of the septal and lateral walls, and only collected in patients in sinus rhythm at the time of their echocardiogram. Mitral regurgitation (MR) was graded numerically: 0, none; 1, trace; 2, mild; 3, mild‐to‐moderate; 4, moderate; 5, moderate‐to‐severe; and 6, severe. LAD was measured in the parasternal long‐axis view. LAVi was measured by the area‐length method.

### 2.3 Endpoints

**Atrial arrhythmia** included all non‐paroxysmal AF, atrial flutter, or focal/multifocal atrial tachycardia during follow‐up; this was assessed by 12‐lead ECGs, ambulatory ECGs, device interrogations, and clinic letters. **Atrial intervention** was a composite of CA and AV node ablation; the latter excludes pacemaker implantation for third‐degree AV block following septal intervention (such as ethanol septal ablation or septal myectomy). **All‐cause mortality** included death from any cause; if the date of death was unavailable, the final clinical contact was used. If an endpoint occurred multiple times (e.g., redo atrial ablation), only the first event was included.

### 2.4 Statistical Analysis

All statistics were performed using Microsoft Excel for Mac 16.86 and IBM SPSS Statistics for Mac 18.0.1.1. Continuous variables were assessed for normality using the Kolmogorov–Smirnov test and thus reported using median [interquartile range]. Categorical variables were reported using percentages, and trends were assessed using McNemar's test. Statistical significance was defined as *p* < 0.05, and was considered borderline if 0.05 ≤ *p* <0.1. Survival analysis was conducted with respect to endpoints, using Cox regression. Univariate analysis was conducted first, and if more than one variable (including either E/e′, LAD or LAVi) was either borderline or significant, multivariate analysis was conducted. Where LAD **or** LAVi were significant on multivariate analysis, ROC analysis was performed for both to compare their discriminative power.

## 3 Results

### 3.1 Sample Characteristics

The final study population comprised 233 patients (median age at entry 53, 73% male), over a median follow‐up of 8 years. Basic demographics and endpoints were available in all patients (i.e., 100%), and echocardiography in > 90% (Table 1).

**TABLE 1:** Sample characteristics for the study population (*n* = 233).

	Index	End of follow‐up	*p* value
**Basic demographics**			
**Age**	53 [43,64]	61 [51,72]	
**Follow‐up, years**	8 [5,10]		
**Male, %**	73		
**BMI**	29 [26,33]		
**Septal intervention, %**	10	17	**< 0.001**
**Device**	23	42	**< 0.001**
**PPM/ICD/CRT**	6/17/<1 (*n* = 1)	11/28/3 (*n* = 8)	**0.002/<0.001/0.016**
**Echocardiography**			
**Septal thickness, mm**	18 [15,20]		
**Peak LVOT gradient, mmHg**	7 [5,22]		
**LVSD, %**	7		
**E/e′**	10 [7,13]		
**LAD, mm**	40 [36,45]		
**LAVi, ml/m** ^2^	69 [56,91]		
**MR, grade**	2 [1,2]		
**Endpoints**			
**Atrial arrhythmia**	11	31	**< 0.001**
**Catheter ablation**		5	
**AV node ablation**		6	
**All‐cause mortality**		12	

*Note*: Bold writing was just the writing style for the group.

There was a significant increase in septal intervention and atrial arrhythmia (both *p *< 0.001); device implantation increased both overall (*p* < 0.001) and for each device type (excluding patients who had a pacemaker for third‐degree AV block after septal intervention). The most common device was an ICD, followed by a pacemaker and then cardiac resynchronization therapy. Twelve percent died over follow‐up. Atrial intervention was uncommon, with only 22 patients (9%) having CA or AV node ablation (three patients had both).

### 3.2 Survival Analysis

#### 3.2.1 Atrial Arrhythmia

**FIGURE 2:**
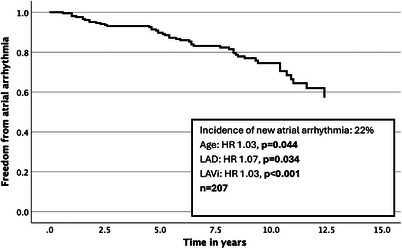
Survival curve with respect to atrial arrhythmia.

The prevalence of new non‐paroxysmal atrial arrhythmia was 22%; this excludes patients who had a history of atrial arrhythmia at index, so it was derived from 89% of the sample (*n* = 207). The median age was 67, and 78% were male. The overall prevalence was 31% (Figure 2).

**FIGURE 3:**
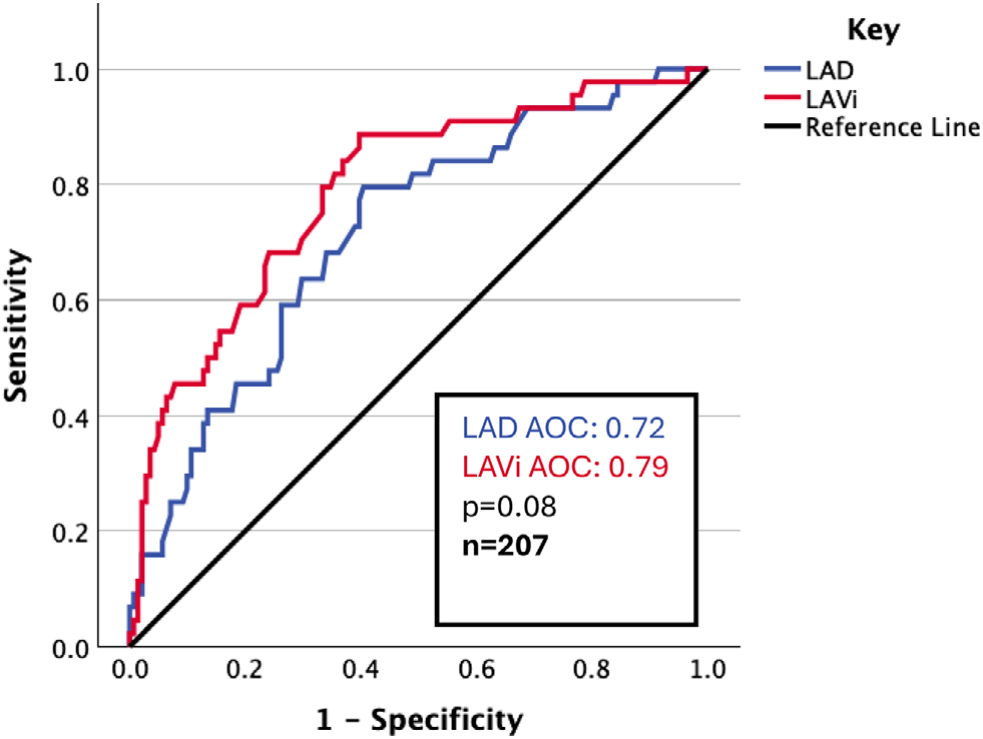
ROC curve for LAD and LAVi with respect to atrial arrhythmia.

On univariate analysis (Table 2), age, E/e', LAD and LAVi′ were significantly associated. On multivariate analysis (Table 2), age (HR 1.03, *p* = 0.044), LAD (HR 1.07, *p* = 0.034) and LAVi (HR 1.03, p < 0.001) were significant; E/e′ was not significant. On ROC analysis, LAD and LAVi had an AOC of 0.72 and 0.79, respectively, but this difference was not statistically significant (Figure 3).

**TABLE 2:** Cox regression with respect to atrial arrhythmia (*n *= 207).

Variable	Univariate		Multivariate	
	HR [95% CI]	*p* value	HR [95% CI]	*p* value
Age, /1 year	1.05 [1.02,1.07]	**< 0.001**	1.03 [1.00,1.06]	**0.044**
Male sex	1.47 [0.73,2.96]	0.28		
BMI, /1 unit	1.02 [0.97,1.08]	0.37		
Septal thickness, mm	1.00 [0.94,1.06]	1.00		
Peak LVOT gradient, mmHg	1.00 [1.00,1.01]	0.49		
LVSD present	2.21 [0.78,6.22]	0.13		
E/e′, /1 unit	1.07 [1.01,1.13]	**0.015**	1.00 [0.93,1.08]	0.91
LAD, /1 mm	1.16 [1.10,1.22]	**< 0.001**	1.07 [1.01,1.15]	**0.034**
LAVi, /1 mL/m^2^	1.04 [1.03,1.05]	**< 0.001**	1.03 [1.01,1.04]	**< 0.001**
MR, /1 grade increase	1.18 [0.94,1.50]	0.15		

*Note*: Bold writing was just the writing style for the group.

#### 3.2.2 Atrial Intervention

**FIGURE 4:**
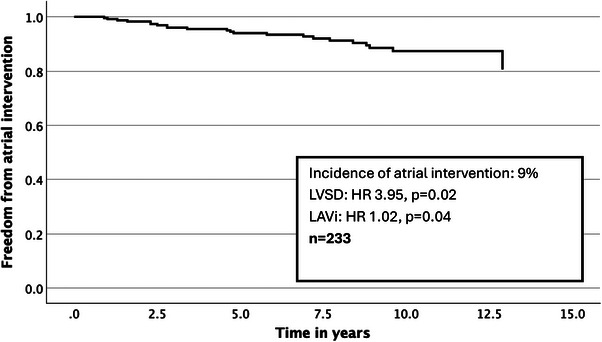
Survival curve with respect to atrial intervention.

The prevalence of atrial intervention was 9% (*n* = 22). This importantly comprised both CA and AV node ablation. The median age was 58, and 86% were male (Figure 4).

**FIGURE 5:**
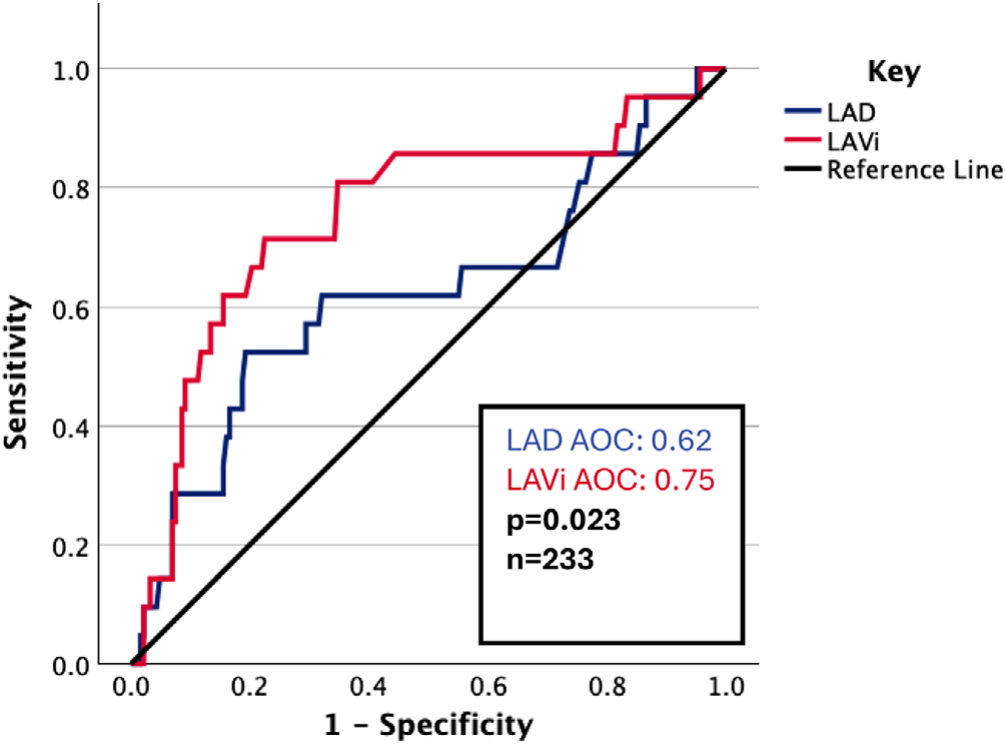
ROC curve for LAD and LAVi with respect to atrial intervention.

On univariate analysis (Table 3), LVSD and LAVi were significantly associated; BMI was borderline. On multivariate analysis (Table 3), LVSD (HR 3.95, *p* = 0.02) and LAVi (HR 1.02, *p* < 0.001) were significant. LAD was not significant, in contrast to the atrial arrhythmia endpoint. LVSD was significant, noting again that this endpoint includes AV node ablation. BMI was not significant. On ROC analysis, LAD and LAVi had an AOC of 0.62 and 0.75, respectively, which was a statistically significant difference (*p* = 0.023) (Figure 5).

**TABLE 3:** Cox regression with respect to atrial intervention (*n* = 233).

Variable	Univariate		Multivariate	
	HR [95% CI]	*p* value	HR [95% CI]	*p* value
Age, /1 year	1.01 [0.98,1.04]	0.68		
Male sex	2.23 [0.66,7.54]	0.20		
BMI, /1 unit	0.92 [0.85,1.01]	0.08	0.94 [0.86,1.03]	0.16
Septal thickness, /1 mm	0.97 [0.87,1.07]	0.49		
Peak LVOT gradient, mmHg	0.98 [0.96,1.01]	0.15		
LVSD present	3.66 [1.23,10.89]	**0.02**	3.95 [1.23,12.71]	**0.02**
E/e′, /1 unit	1.01 [0.92,1.11]	0.84		
LAD, /1 mm	1.08 [1.02,1.14]	**0.01**	1.01 [0.93,1.11]	0.77
LAVi, /1 mL/m^2^	1.02 [1.01,1.03]	**<0.001**	1.02 [1.00,1.03]	**0.04**
MR, /1 grade increase	1.25 [0.87,1.78]	0.23		

*Note*: Bold writing was just the writing style for the group.

#### 3.2.3 All‐Cause Mortality

**FIGURE 6:**
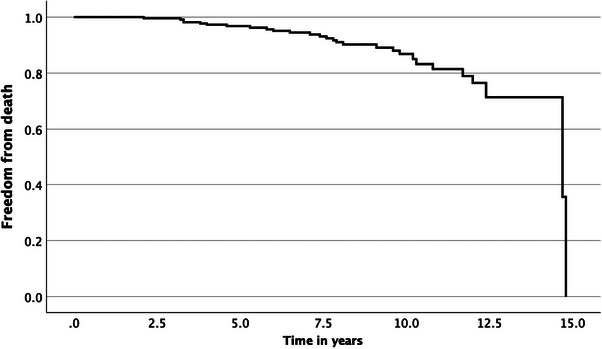
Survival curve with respect to all‐cause mortality.

The prevalence of all‐cause mortality was 12% (*n* = 28). The median age was 71, and 71% were male. On univariate analysis, age, LVSD, and LAD were significantly associated. These all were non‐significant in multivariate analysis (except age, unsurprisingly); LVSD was borderline. Two patients died beyond 14 years of follow‐up, at ages 67 and 69 (Table 4 and Figure 6).

**TABLE 4:** Cox regression with respect to all‐cause mortality (*n* = 233).

Variable	Univariate		Multivariate	
	HR [95% CI]	*p* value	HR [95% CI]	*p* value
Age, /1 year	1.06 [1.02,1.10]	**0.001**	1.06 [1.02,1.10]	**0.002**
Male sex	1.44 [0.54,3.83]	0.46		
BMI, /1 unit	0.97 [0.90,1.05]	0.51		
Septal thickness, /1 mm	1.00 [0.92,1.08]	0.96		
Peak LVOT gradient, mmHg	1.00 [0.98,1.01]	0.65		
LVSD present	2.95 [1.01,8.60]	**0.048**	2.73 [0.93,8.05]	0.068
E/e′, /1 unit	1.02 [0.94,1.12]	0.60		
LAD, /1 mm	1.06 [1.00,1.12]	**0.043**	1.04 [0.98,1.11]	0.16
LAVi, /1 mL/m^2^	1.01 [0.99,1.02]	0.37		
MR, /1 grade increase	0.87 [0.62,1.22]	0.41		

*Note*: Bold writing was just the writing style for the group.

## 4 Discussion

The current study included 233 patients over a median 8 years of follow‐up. It was based in a single tertiary center in the North East of England, with an established cardiothoracic transplant program. Locally, there is greater socioeconomic deprivation [27] and less ethnic diversity [28], both having implications on cardiovascular risk in general [29, 30]. As is common in general cardiovascular and hypertrophic cardiomyopathy research, there was a male predominance.

Otherwise, demographics were largely comparable to previous reports. There was perhaps a greater prevalence of non‐paroxysmal atrial arrhythmia and device implantation, at 31% and 42%, respectively. The atrial arrhythmia may be explained by including all atrial arrhythmia and not only AF. CA occurred in 12 patients, perhaps indicating judicious patient selection.

Similarly, echocardiography was largely comparable, recognizing mitral disease would have been underrepresented (any history of mitral surgery, mitral stenosis, or mitral annular calcification was excluded). The median LAVi was 69 mL/m^2^, but should be interpreted with caution, as area‐length may overestimate volumes [24, 25].

LAVi was an independent predictor of non‐paroxysmal atrial arrhythmia and atrial intervention, and LAD was only the former. LAVi showed superior discriminative power compared to LAD, in predicting atrial intervention (AOC 0.75 vs 0.62, respectively, *p* = 0.023), but was borderline for arrhythmia (AOC 0.79 vs 0.71, *p* = 0.08). It is possible larger sample sizes may contribute to further statistically significant findings; that said, the sample size was comfortably higher than power calculations. There was extensive adjustment for covariates. E/e′ was not significant in any multivariate analyses. No echocardiographic variable was associated with all‐cause mortality in multivariate analysis.

This helps contextualize the clinical value of left atrial size assessment in patients with HCM. Although LAD is enshrined widely in the evidence base and therefore available risk scores, LAVi may be more reliable. Interestingly, peak LVOT gradient and septal thickness were not significant in any analyses. It is difficult to elucidate why, but it may be reflective of a more proactive clinical routine in a tertiary setting, influencing the underlying phenotype. LVSD was associated with atrial intervention, perhaps accounting for patients with dual indication for device therapy, as per general cardiology guidelines for severe LV impairment [31].

Future work should use larger samples to validate different measures of left atrial size, as clinical practice varies. The authors suggest that these should include LAD, left atrial area, and LAVi by the biplane method (not area‐length).

## 5 Conclusion

In a single UK tertiary center, LAVi was an independent predictor of atrial arrhythmia and intervention; LAD was only predictive of the former. LAVi demonstrated superior discriminative power in predicting interventions than LAD. E/e′ was not associated with any outcome in multivariate analysis. Furthermore, no echocardiographic variable was associated with all‐cause mortality, including LVOT gradient and septal thickness. Future work should clarify which measures of left atrial size are most appropriate in clinical practice.

## Funding

The authors have nothing to report.

## Ethics Statement

The chair of the ethics committee confirmed formal REC approval was not required. Registered on the local Clinical Effectiveness Database.

## Conflicts of Interest

The authors declare no conflicts of interest.

## Data Availability

The data that support the findings of this study are available from Newcastle upon Tyne NHS Foundation Trust. Restrictions apply to the availability of these data, which were used under license for this study. Data are available from the author(s) with the permission of Newcastle upon Tyne NHS Foundation Trust.
